# Disease duration of progression is helpful in identifying isolated bulbar palsy of amyotrophic lateral sclerosis

**DOI:** 10.1186/s12883-021-02438-8

**Published:** 2021-10-22

**Authors:** Huagang Zhang, Lu Chen, Jinzhou Tian, Dongsheng Fan

**Affiliations:** 1 Department of Neurology, Peking University Third Hospital, No. 49, North Garden Road, Haidian District, Beijing, 100191 China; 2 Beijing Municipal Key Laboratory of Biomarker and Translational Research in Neurodegenerative diseases, Beijing, China; 3 Neurology Centre, Dongzhimen Hospital, Beijing University of Chinese Medicine, Beijing, China; 4 Key Laboratory for Neuroscience, National Health Commission/Ministry of Education, Peking University, Beijing, China

**Keywords:** Bulbar palsy, Amyotrophic lateral sclerosis, Survival, Motor neuron, Prognosis

## Abstract

**Background:**

Compared with typical bulbar onset amyotrophic lateral sclerosis (ALS), isolated bulbar palsy (IBP), an often under-understood variant of ALS, is characterized by symptoms confined to bulbar region for extended periods and relative preservation of limb and ventilation function. To find a cutoff value of disease duration that can distinguish IBP from typical bulbar onset ALS well, the association of survival with disease progression in bulbar onset ALS patients was analyzed.

**Methods:**

Clinical data of bulbar onset ALS patients were collected from January 2009 to December 2013. The duration from bulbar onset to first significant limb involvement was analyzed by a cutoff point analysis with maximally selected log-rank statistics and dichotomized to categorize patient outcomes. The patients were divided into two groups, the IBP and typical bulbar onset ALS groups, according to the cutoff value. Clinical features were compared.

**Results:**

115 bulbar onset ALS patients were recruited, and the duration from bulbar onset to first significant limb involvement was associated with survival (*P* < 0.001). The cutoff duration was 20 months. 19 patients were identified as IBP and 96 patients as typical bulbar onset ALS using 20 months as the cutoff duration. Female was more common, limb weakness was less frequent and pure upper motor neuron (UMN) bulbar signs were more frequent in the IBP group than in the typical bulbar onset ALS group (*P* = 0.047; *P* = 0.004; *P* = 0.031). The median survival time of the IBP group was significantly longer than that of the typical bulbar onset ALS group (64 months and 26 months, respectively; *P* < 0.001).

**Conclusions:**

A cutoff duration of 20 months from bulbar onset to first significant limb involvement may be used to specifically distinguish IBP from typical bulbar onset ALS. IBP was characterized by female predominance, relative preservation of limb function, more pure UMN bulbar signs and a relatively benign prognosis.

## Background

The initial symptoms of typical bulbar onset amyotrophic lateral sclerosis (ALS) include dysarthria or dysphagia, and the development of progressive limb symptoms and signs in short-term generally portends a poor prognosis [1–4].

However, a small group of bulbar onset ALS patients do not progress as rapidly as typical bulbar onset ALS patients do. Dysarthria or dysphagia emerges insidiously and progresses slowly in these patients who appear to have a relatively benign prognosis [5, 6]. Compared with typical bulbar onset ALS, isolated bulbar palsy (IBP), an often under-understood variant of ALS, is characterized by symptoms confined to bulbar region for extended periods and relative preservation of limb and ventilation function [5, 6]. Consequently, it is important to distinguish IBP from typical bulbar onset ALS for prognosis prediction, patient care and even treatment options.

According to the limited number of reports on IBP that are currently available, bulbar onset ALS patients who had no significant limb involvement or evidence of progression over the initial disease course of six months were diagnosed as IBP [5]. Typical bulbar onset ALS was diagnosed if bulbar onset patients developed progressive limb symptoms and signs within the first six months [5, 6]. The cutoff value of six months was proposed mainly based on clinical experience and is not generally accepted.

The defining feature of IBP compared with typical bulbar onset ALS is relative isolation of clinical symptoms and signs to the bulbar musculature for an extended period, as well as longer survival [5, 6]. Here, we analyzed the association of survival and disease progression in bulbar onset ALS patients and tried to find a cutoff value of disease duration that can distinguish IBP from typical bulbar-onset ALS well.

## Materials and methods

### Patients

The clinical data of all patients with sporadic ALS were reviewed who were registered to Department of Neurology of PUTH from January 2009 to December 2013. All patients were diagnosed according to the Airlie House diagnostic criteria by neurologists from Department of Neurology of PUTH who were expert in diagnosis and treatment of ALS [7]. The study group collected elementary demographic information and clinical data from all patients during first visit in PUTH. Each patient diagnosed with bulbar onset ALS who had complaints of an insidious onset of dysarthria, dysphagia or both was selected. Detailed inspection, including magnetic resonance imaging (MRI) of the central nervous system, electromyography (EMG), neurophysiological and serum autoantibody tests were used to exclude alternative diagnoses. Patients with familial and juvenile ALS were excluded from the study. Spinal and bulbar muscular atrophy (SBMA) was excluded with genetic test for exon 1 of androgen receptor gene if SBMA was difficult to clinically distinguish from bulbar onset ALS [8].

Each patient underwent a follow-up evaluation by telephone every 3 or 6 months after the first visit till December 2016. Revised ALS functional rating scale (ALSFRS-R) was assessed by telephone at each follow-up. Whether a patient used noninvasive ventilation (NIV) was recorded in the sub-score (Respiration 3-respiratory insufficiency) of the respiratory items of ALSFRS-R. The sub-score of Respiration 3-respiratory insufficiency indicated that a patient used NIV when the sub-score was 1 to 3. ‘Use of riluzole’ was defined as treatment with riluzole (50 mg) twice a day for longer than 2 weeks [4]. Death or tracheotomy was predefined as the primary outcome measure.

The duration from bulbar onset to first significant limb involvement (FSLI) is crucial for distinguishing IBP from typical bulbar onset ALS. Significant limb involvement was defined as symptomatic limb weakness that contributed to the clinical presentation [5]. The neurologists of the study group judged whether a patient with bulbar onset ALS had significant limb involvement on the basis of the clinical history combined with physical examination during the patient’s first visit in our department. If no significant limb involvement was found, symptomatic limb weakness would be obtained by asking the patients through regular telephone follow-up, as recorded in sub-score of ALSFRS-R. The duration and survival time were calculated on the basis of the clinical history, physical examination and follow-up findings. Patients who were lost to follow-up or did not know the exact time of FSLI were excluded.

### Investigations

The experienced neurologists from the study group simultaneously assessed revised ALS functional rating scale (ALSFRS-R) and forced vital capacity (FVC) for each patient during patients’ first visit in PUTH. The aggregate score of ALSFRS-R was 48.

FVC was tested by professionals in the pulmonary function examination room of PUTH. Patients were instructed to fasten the blowing nozzle with mouth to ensure no air leakage during the detection process. Patients inhaled completely, then exhaled quickly and forcefully until fully exhaled as instructed. Each patient completed at least three qualified expiration movements and the largest value was identified as FVC. FVC values were expressed as percentages of the predicted values [9]. FVC values less than 80% were considered abnormal, representing ventilation dysfunction [9]. Diseases involving the cardiopulmonary system were excluded.

### Statistical analysis

Cox regression was used to analyze the influence of factors on survival. The duration from bulbar onset to FSLI was analyzed as a continuous variable and dichotomized to distinguish IBP from typical bulbar onset ALS. A cutoff point analysis was performed by maximally selected log-rank statistics using R software v. 3.4.2 (packages: maxstat) [10]. Different durations in months were examined as candidate cutoff points, and the cutoff duration that best separated patient outcomes was chosen on the basis of the maximum relative risk and minimum *P* value instead of an arbitrary selection.

All bulbar onset ALS patients were divided into two groups, the IBP and typical bulbar onset ALS groups, according to the cutoff value. Differences between the IBP and typical bulbar onset ALS groups in elementary clinical features, ALSFRS-R and FVC first evaluated in PUTH and survival were analysed by SPSS 18.0 software for Windows (SPSS Inc., Chicago, IL, USA). Whether the data were normally distributed was determined using Kolmogorov-Smirnov test. Quantitative data that were normally distributed were expressed as mean ± standard deviations (SDs), and quantitative data that were non-normally distributed were expressed as the medians and ranges (minimums, maximums) [11]. Differences in categorical variables were analysed by *χ*^2^ test or Fisher’s exact test, and continuous variables were evaluated by independent samples *t*-test or Mann-Whitney *U*-test depending on the parametric or nonparametric nature of the data [11]. Kaplan-Meier analysis and log-rank test were used to compare survival between the two groups [11]. A value of *P* < 0.05 (two-sided) was considered statistically significant.

## Results

### Patient demographics

From a total of 1177 consecutive sporadic ALS patients, 154 were bulbar onset patients. Among these patients, 39 were excluded, including 33 who were lost to follow-up and 6 who did not know the exact time of FSLI, and the remaining 115 patients (66 males, 49 females, mean age 57.8 ± 10.3 years, range 34–80 years) were retrospectively recruited.

The 115 bulbar onset ALS patients exhibited a mean onset age of 56.9 ± 10.2 years (range 34–79 years), a median disease course of 12 (2, 43) months from the initial symptoms and a mean ALSFRS-R score of 39.8 ± 6.0 (range 18–48) at first visit in our department. The median duration from bulbar onset to FSLI was 9 (1, 39) months. Next to bulb, upper limb, lower limb or ipsilateral upper and lower limbs almost at the same time were first significantly involved in 100, 7 and 4 patients respectively. No limbs were involved in 4 patients till they suffered death or tracheotomy. Only 34 (29.6%) and 23 (20.0%) patients used riluzole and noninvasive ventilation (NIV), respectively. A total of 111 patients suffered primary outcome events by December 2016, and the median survival time was 29 (11, 83) months.

### Cutoff point of duration from bulbar onset to FSLI

**Fig. 1:**
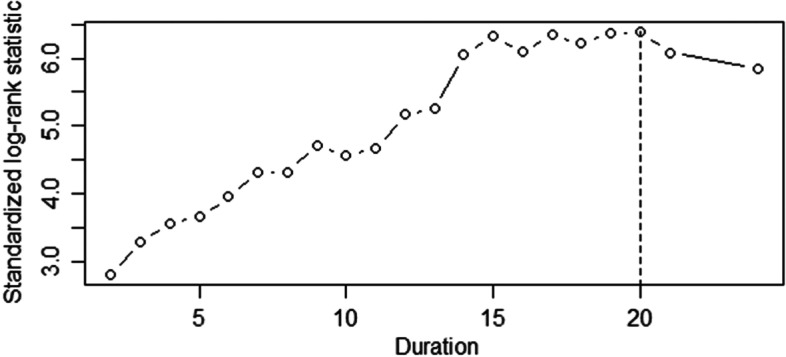
Cutoff point of duration from bulbar onset to FSLI in 115 bulbar onset ALS patients. The Duration in months from bulbar onset to FSLI in 115 bulbar onset ALS patients was analysed as a candidate for the cutoff point by maximally selected log-rank statistics according to a maximum relative risk and minimum *P* value. The cutoff duration was 20 months. FSLI = first significant limb involvement; ALS = amyotrophic lateral sclerosis; IBP = isolated bulbar palsy

Cox regression was used to analyze the influence of factors on survival, including gender, onset age, the duration from bulbar onset to FSLI, ALSFRS-R at first visit in our department, use of riluzole and NIV (Table 1). The duration from bulbar onset to FSLI had the most significant influence on survival time (*P* < 0.001, HR = 0.912, 95% CI = 0.886–0.939). The other five factors had no significant influence on survival time. The 115 bulbar onset ALS patients were divided into two groups, the IBP and typical bulbar onset ALS groups, according to the duration from bulbar onset to FSLI and the cutoff value determined by maximally selected log-rank statistics. The cutoff duration was 20 months (Fig. 1). Therefore, IBP was defined as patients with a duration ≥20 months from bulbar onset to FSLI, whereas typical bulbar onset ALS patients had a duration < 20 months.

**Table 1:** Multivariate Cox regression analysis of influence of factors on survival in bulbar onset ALS patients

Variable	HR	95% CI	***P***
Gender			
Famale	1.000		
Male	0.721	0.470–1.106	0.134
Onset age (year)	1.018	0.998–1.038	0.074
Duration (month)	0.912	0.886–0.939	< 0.001
ALSFRS-R	0.986	0.955–1.018	0.380
Use of riluzole			
No	1.000		
Yes	1.170	0.755–1.812	0.483
Use of NIV			
No	1.000		
Yes	0.951	0.577–1.568	0.844

*ALS* amyotrophic lateral sclerosis, *Duration* duration from bulbar onset to first significant limb involvement, *ALSFRS-R* Revised ALS Functional Rating Scale, *NIV* noninvasive ventilation

### Clinical characteristics

According to the above definition, 19 patients were identified as having IBP (7 males, 12 females, mean onset age 56.9 ± 9.7 years, median disease course 25 (10, 43) months, mean ALSFRS-R score 41.1 ± 3.1) and 96 patients were categorized as having typical bulbar onset ALS (59 males, 37 females, mean onset age 56.9 ± 10.3 years, median disease course 10 (2, 39) months, mean ALSFRS-R score 39.6 ± 6.4). The clinical characteristics of 115 bulbar onset ALS patients at first visit in our department were showed in Table 2. There were 5 (26.3%) and 4 (21.1%) patients using riluzole and NIV in the IBP group, respectively. In the other group, there were 29 (30.2%) and 19 (19.8%) patients using riluzole and NIV, respectively. Female was more common, and disease course was longer in the IBP group than in the typical bulbar onset ALS group (*P* = 0.047; *P* < 0.001), and there were no significant differences in onset age, ALSFRS-R score, the percentages of patients using riluzole or NIV in either group.

**Table 2:** Clinical characteristics of 115 patients with bulbar onset ALS

Groups	Gender, ***n*** (male: female)	Onset age (years)	Course (months)	ALSFRS- R score	Limb Weakness, n (%)	Brisk tendon reflex, n (%)	Babinski’s sign, n (%)	Hoffmann’s/ Rossolimo’s sign, n (%)	Pure UMN bulbar signs, n (%)	Pure LMN bulbar signs, n (%)
**IBP**(*n = 19*) **Typical bulbar onset ALS** (*n = 96*) **Statistics** ***P***	7:12 (0.6:1)	56.9 ± 9.7	25(10,43)	41.1 ± 3.1	6(31.6)	13(68.4)	2(10.5)	5(26.3)	3(15.8)	3(15.8)
	59:37 (1.6:1)^*^	56.9 ± 10.3	10(2,39)^*^	39.6 ± 6.4	64(66.7)^*^	71(74.0)	30(31.3)	42(43.8)	2(2.1)^*^	8(8.3)
	3.930^†^	0.024^‡^	5.271^§^	1.582^‡^	8.198^†^	0.247^†^	3.392^†^	1.995^†^	–	–
	0.047	0.981	< 0.001	0.119	0.004	0.619	0.066	0.158	0.031	0.387

Values are presented as *n* (%), mean ± SD or median (minimum, maximum). ^*^P value for contrast of variates between patients with IBP and typical bulbar onset ALS; *P* < 0.05, as compared with patients with IBP. ^†^*χ*^2^ values; ^‡^*t* values; ^§^*Z* values. *ALS* Amyotrophic lateral sclerosis, *IBP* Isolated bulbar palsy, *ALSFRS-R* Revised ALS Functional Rating Scale, *UMN* Upper motor neuron, *LMN* Lower motor neuron, − = Not applicable, *SD* Standard deviation

We examined the sub-score of the bulbar items of ALSFRS-R score (Speech; Swallowing) of the 115 bulbar onset ALS patients at first visit in our department. The sub-scores of all 19 IBP patients were less than 4 points in both speech and swallowing, so the proportion of dysarthria was the same as that of dysphagia in IBP patients. In the typical bulbar onset ALS group, only one patient scored 4 points in speech and three patients scored 4 points in swallowing. The sub-scores of the remaining typical bulbar onset ALS patients were less than 4 points in both speech and swallowing (dysarthria 99.0%, dysphagia 96.9%). There was no significant difference in the abnormal rate of speech or swallowing in either group (*P* = 1.000; *P* = 1.000).

Clinically, limb weakness was less frequent, and pure upper motor neuron (UMN) bulbar signs, including palmomental reflex, sucking reflex and jaw jerk, were more frequent in the IBP group than in the typical bulbar onset ALS group (*P* = 0.004; *P* = 0.031) (Table 2). Limb weakness developed significantly later in IBP patients than in typical bulbar onset ALS patients. The median duration from bulbar onset to the FSLI was 28 (20, 39) and 7 (1, 19) months (*P* < 0.001) for the IBP and typical bulbar onset ALS groups, respectively. There were no significant differences in brisk tendon reflex; Babinski’s sign; Hoffmann’s or Rossolimo’s sign; pure lower motor neuron (LMN) bulbar signs, including tongue amyotrophy or fasciculation; or neurogenic damages, as assessed by EMG, in the tongue, sternocleidomastoid or upper trapezius muscles.

### FVC

FVC was examined in 7 IBP patients (3 males, 4 females, median disease course 19 (12, 35) months) and 31 typical bulbar onset ALS patients [21 males, 10 females, median disease course 8 (2, 33) months] at first visit in PUTH. The mean FVC value was 80.1 ± 9.6% (range 63–89.4%) of the predicted value in IBP patients and 83.2 ± 15.3% (range 49–109%) of the predicted value in typical bulbar onset ALS patients. Three patients in the IBP group and 13 patients in the other group had a FVC of < 80%. No significant differences were noted in the mean FVC value or the incidence of FVC being < 80%, but there was a difference in disease course (*P* = 0.001) between the two groups. The remaining 77 patients could not or refused to complete the FVC assessment because of weakness or amyotrophy of the bulbar muscles or other reasons, such as there being an inspection fee or the time of the appointment being inconvenient.

### Survival

**Fig. 2:**
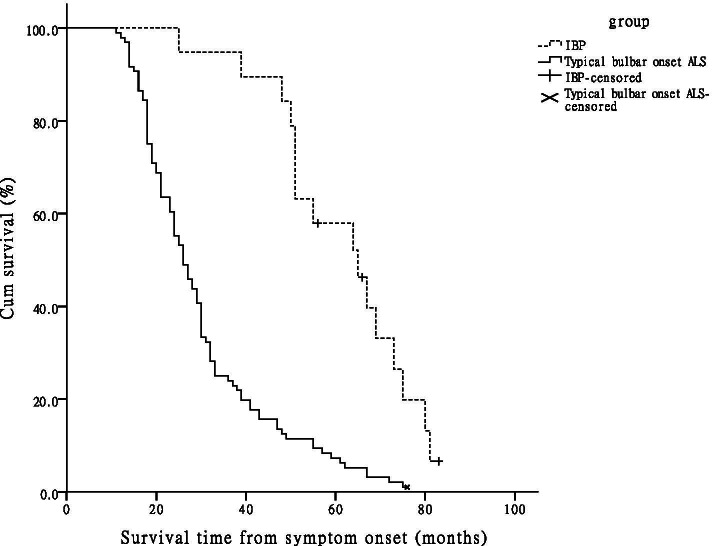
Kaplan-Meier survival curves of 115 bulbar onset ALS patients according to the phenotype. When 20 months was used as the cutoff duration, 19 patients were identified as having IBP, and 96 patients were identified as having typical bulbar onset ALS. Kaplan-Meier analysis and log-rank test showed that the median survival time of the IBP patients was significantly longer than that of the typical bulbar onset ALS patients (*P* < 0.001). ALS = amyotrophic lateral sclerosis; IBP = isolated bulbar palsy

A smaller proportion of IBP patients than of typical bulbar onset ALS patients suffered primary outcome events within the follow-up period (IBP, 16, 84.2%; typical bulbar onset ALS, 95, 99.0%). The median survival time was 64 (25, 83) months in the IBP group and 26 (11, 76) months in the typical bulbar onset ALS group. The Kaplan-Meier curves of the two groups were shown in Fig. 2, and Log-rank test showed that the median survival time of IBP patients was significantly longer than that of typical bulbar onset ALS patients (Log rank = 27.679, *P* < 0.001). Ninety-six typical bulbar onset ALS patients were divided into three groups according to the duration from bulbar onset to FSLI: duration ≤6 months, from 7 to 12 months and from 13 to 19 months. There were 46 (47.9%), 28 (29.2%) and 22 (22.9%) patients in each group, and 31 (67.4%), 12 (42.9%) and 6 (27.3%) patients with survival time ≤ 26 months in each group, respectively.

## Discussion

IBP is an uncommon regional variant of ALS that seems to progress more slowly than typical bulbar onset ALS does [5, 6]. We studied the association between survival and duration from bulbar onset to FSLI in bulbar onset ALS patients and found that the duration could be dichotomized by a cutoff duration of 20 months to distinguish IBP from typical bulbar onset ALS. Patients with a duration ≥20 months from bulbar onset to FSLI are more likely to have IBP; in contrast, typical bulbar onset ALS has a duration < 20 months. Therefore, a duration of ≥20 months from bulbar onset to FSLI may help to distinguish IBP from typical bulbar onset ALS.

In prior reports, a cutoff duration of six months for distinguishing IBP from typical bulbar onset ALS was proposed mainly based on clinical experience, with which absence of limb progression over a six-month period was considered to be atypical for bulbar onset ALS [5]. The cutoff duration of 20 months, which was selected according to clinical data and statistical analysis, far exceeds six months. In the present study, 18 of 50 (36.0%) patients in the group with a duration of 7 to 19 months from bulbar onset to FSLI and even 6 of 22 (27.3%) patients in the group with a duration of 13 to 19 months had survival time ≤ 26 months, which was the median survival time in typical bulbar onset ALS patients and is consistent with the findings in prior reports [3]. These patients, who would be diagnosed as IBP according to the prior criteria but had poor prognosis, were diagnosed as typical bulbar onset ALS in accordance with the present cutoff value. The prior cutoff duration of six months might lead to a considerable number of typical bulbar onset ALS being diagnosed as IBP and remarkably reduce the specificity of IBP diagnosis. However, all the patients in this study were from mainland China, and the clinical characteristics and outcomes of Chinese patients with sporadic ALS are different from those of patients from other countries [4]. The cutoff value may vary among individuals of different races and regions.

Prior studies on IBP showed its lower morbidity, which was estimated to be approximately 1–4% of ALS patients [5, 6, 11]. IBP was characterized by a female predominance and respiratory function preserved in a relatively long period. Patients with IBP might be younger or older in onset age than typical bulbar onset ALS and have UMN and/or LMN signs in the bulbar region [5, 6, 11]. Percutaneous endoscopic gastrostomy (PEG) tube placement might be required at an early phase because of dysphagia [5, 6].

A large prospective cohort study showed some characteristics of bulbar onset ALS patients in Chinese mainland. The frequency of bulbar onset ALS (14.0%) was much lower in ALS patients in China than that in some other countries [4]. The ratio of male to female, mean onset age and median survival time of this phenotype were 1.3: 1, 53.3 years and 48 months, respectively [4]. Our study showed that the ratio of male to female, mean onset age and median survival time of bulbar onset ALS patients were 1.3: 1, 56.9 years and 29 months, respectively. The present study also described clinical features of IBP according to a duration of ≥20 months from bulbar onset to FSLI in mainland China. Patients with IBP accounted for 16.5% of bulbar onset ALS patients. There was a female predominance in IBP, which indicates that genetic factors may play a role. However, IBP has been mostly reported as sporadic cases, and genetic susceptibility to IBP needs to be studied further. The disease course and survival time were significantly longer, and the proportion of limb weakness was significantly smaller in the IBP group than in the typical bulbar onset ALS group, which coincide with the viewpoint of slower progression, relative isolation of symptoms in the bulbar region and a relative benign prognosis of IBP [5, 6, 11]. Pure UMN bulbar signs were more frequent in IBP, which may help to clinically distinguish IBP from typical bulbar onset ALS [5]. Some of the results in our study may have differed from those in prior reports because of differences in patients’ races and regions, the sample size among studies and large clinical heterogeneity of ALS, especially in research methods and selection criteria of patients.

ALS is characterized by focal and regional susceptibility in pathophysiologic mechanism, which implies that the region adjacent to onset site is apt to be prior involved [12]. In a recent study, Gromicho et al. found that contiguous spreading was the leading progression pattern in ALS and regional progression of LMN degeneration was most likely contiguous [13]. The 3rd to 5th segments of cervical spinal cord, which innervate diaphragm, are tightly adjoined to medulla oblongata, and LMNs in the two regions may be concurrently impaired in ALS. Consequently, respiratory failure may occur earlier in patients with typical bulbar onset ALS than in patients with IBP [11]. Motor neurons damage in IBP is confined to the bulbar region for extended periods for unknown reasons, which may partially explain the phenomenon of respiratory function preserved in IBP [6, 11]. The present study showed no significant differences in the mean FVC value or the proportion of patients with FVC < 80%, but there was a difference in the disease course between the 7 IBP and 31 typical bulbar onset ALS patients at first visit in our department. If the sample size is increased or FVC is assessed at the same time in the disease course, significant differences in FVC may be revealed in view of the result that the disease course of IBP was notably longer than that of typical bulbar onset ALS and the fact that ALS patients’ respiratory symptoms usually aggravate gradually with the disease course prolonging [1, 2].

Understanding the characteristics of IBP may help to provide important information about the pathogenesis of ALS and guidance for clinical trials and treatment options. In future trials including patients with IBP or other regional variants of ALS, researchers should consider stratifying the patients to reduce the variability in response to therapy owing to the differences in the rate of progression [6].

This study has several limitations. The sample size was comparatively small, especially that of the group who underwent FVC examination. The clinical significance of the study may be limited by selection bias, and multicenter studies are warranted. The lack of cognitive evaluation and information about patients using PEG are two other deficiencies.

## Conclusions

The primary finding of this study was that bulbar onset ALS can be classified as IBP and typical bulbar onset ALS using 20 months as the cutoff duration from bulbar onset to FSLI. IBP appears to constitute a distinct clinical phenotype of ALS with the characteristics of female predominance, symptoms confined to bulbar region for extended periods, relative preservation of limb function, more pure UMN bulbar signs and a relatively benign prognosis. Prospective studies are required to confirm the findings.

## Data Availability

The datasets used and analyzed during the current study are available from the corresponding author on reasonable request.

## Abbreviations

ALS: amyotrophic lateral sclerosis
IBP: isolated bulbar palsy
UMN: Upper motor neuron
PUTH: Peking University Third Hospital
MRI: Magnetic resonance imaging
EMG: Electromyography
SBMA: Spinal and bulbar muscular atrophy
FSLI: First significant limb involvement
ALSFRS-R: Revised ALS functional rating scale
NIV: Noninvasive ventilation
FVC: Forced vital capacity
LMN: Lower motor neuron
PEG: Percutaneous endoscopic gastrostomy
